# Systematic review of pathways for the delivery of allergy services

**DOI:** 10.1136/bmjopen-2016-012647

**Published:** 2017-02-07

**Authors:** Lavanya Diwakar, Carole Cummins, Richard Lilford, Tracy Roberts

**Affiliations:** 1 Health Economics Unit, University of Birmingham, Birmingham, UK; 2 Institute of Applied Health Research, University of Birmingham, Birmingham, UK; 3 Population Evidence and Technologies Unit, Warwick Medical School, University of Warwick, Coventry, UK

**Keywords:** Allergy Services, PRIMARY CARE, Secondary Care, Systematic review, Scoping review

## Abstract

**Objectives:**

The incidence and prevalence of allergies worldwide has been increasing and allergy services globally are unable to keep up with this increase in demand. This systematic review aims to understand the delivery of allergy services worldwide, challenges faced and future directions for service delivery.

**Methods:**

A systematic scoping review of Ovid, EMBASE, HMIC, CINAHL, Cochrane, DARE, NHS EED and INAHTA databases was carried out using predefined inclusion and exclusion criteria. Data on the geographical region, study design and treatment pathways described were collected, and the findings were narratively reported. This review followed the Preferred Reporting Items for Systematic Reviews and Meta-analyses (PRISMA) guidelines.

**Results:**

205 publications were screened and 27 selected for review. Only 3 were prospective studies, and none included a control group. There were no eligible publications identified from North America, Africa, Australia and most parts of Asia. Most publications relate to allergy services in the UK. In general, allergy services globally appear not to have kept pace with increasing demand. The review suggests that primary care practitioners are not being adequately trained in allergy and that there is a paucity of appropriately trained specialists, especially in paediatric allergy. There appear to be considerable barriers to service improvement, including lack of political will and reluctance to allocate funds from local budgets.

**Conclusions:**

Demand for allergy services appears to have significantly outpaced supply. Primary and secondary care pathways in allergy seem inadequate leading to poor referral practices, delays in patient management and consequently poor outcomes. Improvement of services requires strong public and political engagement. There is a need for well-planned, prospective studies in this area and a few are currently underway. There is no evidence to suggest that any given pathway of service provision is better than another although data from a few long-term, prospective studies look very promising.

**Strengths and limitations of this study**

- The literature review was carried out using eight major databases and reporting followed the PRISMA guidelines.
- This is comprehensive review of all the published reports and journal articles on allergy services.
- No eligible publications were identified from large geographical areas such as North America, Africa, Australia and most of Asia; most publications were UK based.
- Service pathways for allergy and eczema were considered in the review.

## Introduction

The incidence and prevalence of allergic diseases has been steadily increasing globally.^1^ It is recognised that there has been an increase in the prevalence of allergies in children and young adults with each passing decade.^2^ Despite this increasing need, allergy services have not improved worldwide.^3^ It is now well established that developed countries bear a higher burden of allergic disease.^1^ ^4–6^ However, services rendered to the affected individuals in these higher income countries remain inadequate with deficiencies in primary and secondary care provision.^3^ ^7^ The picture is similar across many countries with long waiting times for specialist appointments and wide heterogeneity in provision of primary care and specialist services.^7^ ^8^ In addition, the growing incidence of serious allergic manifestations such as anaphylaxis^9–12^ as well as that of individuals with multiple, complex allergies^13^ has prompted calls for improved services worldwide.^3^ ^13^

The UK has one of the highest rates of allergy and related diseases in the western hemisphere^1^ with a steady increase in the prevalence, severity and complexity of allergic disease in the last two to three decades.^2^ ^14–17^ It is estimated that 30% of all adults and 40% of children in the UK will be affected by allergy-related conditions.^18^ Nevertheless, allergy services have remained ‘woefully poor’^18^ with very limited and patchy specialist service availability. This shortfall in service availability and the inherent heterogeneity of limited available services has been the focus of multiple expert body reviews in the UK, which have called for increased investment in allergy management and for reorganisation of allergy services.^18–22^

One of the major barriers to service planning in allergy is the lack of political engagement and reluctance to allocate funds from the local budget for improving allergy services.^23^ ^24^ Allergy is not generally perceived as a serious condition with major implications for health and quality of life. There is a growing body of evidence to the contrary, however. It is now established that children with food allergies are more anxious than those with insulin-dependent diabetes and tend to have overprotective and very anxious parents.^25^ This is also true of adolescents with a history of anaphylaxis.^26^ In addition, the costs of allergies can be considerable. Allergy and related conditions are estimated to cost the UK NHS about £1 billion per year.^27^ Productivity losses associated with allergic rhinitis in the USA were higher than those due to stress, migraine and depression.^28^ Studies have shown that effective allergy services can not only improve quality of life, but can also be cost-saving.^29^ ^30^ Hence, there is an urgent need to impress on policymakers the importance and wisdom of investing in the improvement of allergy services.

There is currently no agreement on how allergy services should be structured. In the UK and Europe, Primary Care Physicians – known as GPs or General Practitioners in the UK – (PCPs) diagnose and manage the majority of individuals with allergies^7^ whereas in Australia and the USA, specialist services provide the bulk of allergy care.^8^ Allergy service delivery by non-clinician practitioners such as pharmacists and dieticians, while possible, is not optimally used.^22^ Various pathways have been suggested and are being tested.^23^ ^31^ ^32^ However, it is not yet clear whether any particular model of service delivery may be preferable to the others.

The aim of this systematic review is to assess published approaches to allergy service delivery. The objective is to identify and appraise these publications to gain an understanding of the advantages as well as challenges associated with these service pathways; and also to explore current ideas regarding the future direction for these services.

## Methods

The Preferred Reporting Items for Systematic Reviews and Meta-analyses (PRISMA) guidelines were followed in conducting this systematic scoping review. The PRISMA checklist is supplied as online supplementary file S1.

10.1136/bmjopen-2016-012647.supp1 supplementary file 1

### Data sources and search strategy

A systematic search of the literature was carried out to identify articles related to allergy service pathways in humans. Search terms included allergy, eczema, care, service and pathway (see online supplementary file S2). MEDLINE, EMBASE, HMIC, CINAHL, Cochrane, DARE, NHS EED and INAHTA websites were searched for the purposes of this review. Searches included publications indexed until the 4th of October 2016. In order for the MEDLINE searches to be relevant, we stipulated that two papers selected a priori^3^ ^33^ should be identified in the search. References within the publications identified as relevant were individually examined to identify more articles of interest. Publications citing the chosen articles were also carefully examined for relevance.

10.1136/bmjopen-2016-012647.supp2 supplementary file 2

### Selection of literature

After discarding duplicates, the title and abstract of the articles were examined for relevance. Where these were not informative, the full text of the publication was reviewed. Articles were included for review if they discussed pathways for the delivery of allergy or eczema services. Publications which reported opinions, conference abstracts, case reports or case series were excluded. Non-English language articles were not included in the review. Asthma service pathways were also not considered. One of the researchers (LD) carried out the searches with help and advice from an information specialist from the University of Birmingham. LD screened all the articles as per the predetermined criteria. A total of 50% of the unselected articles (25% each) were reviewed independently by two of the coauthors (TR and CC). Disagreements, if any, were resolved through discussion and consensus.

**Figure 1:**
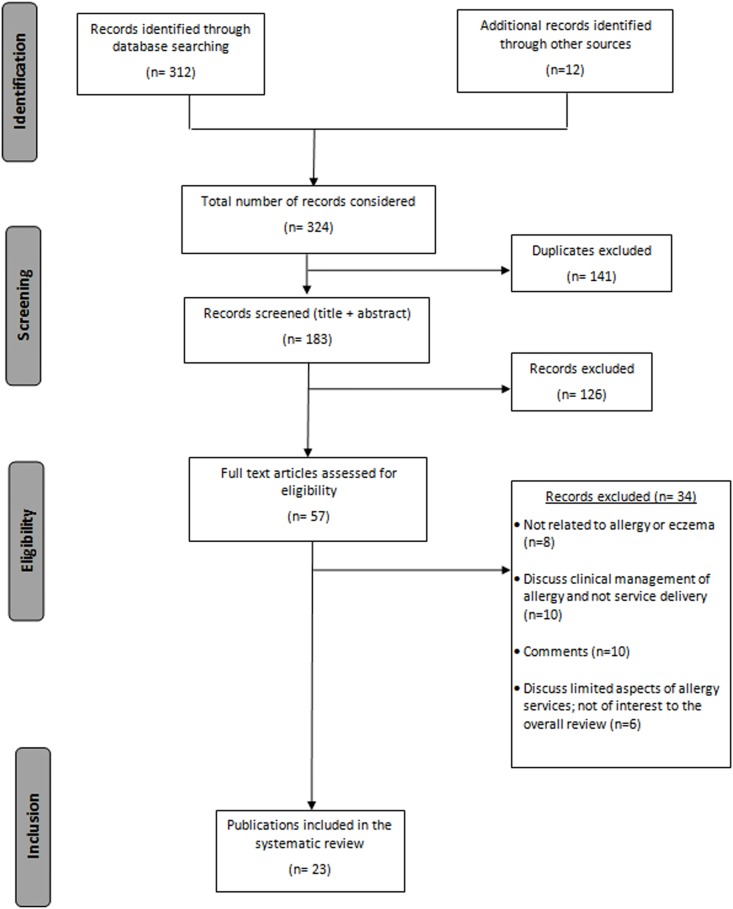
Flow diagram showing the stages involved in choosing eligible publications for the systematic review (based on the PRISMA recommendations).

The PRISMA flow chart for selection of articles is shown in figure 1.

### Data extraction and analysis

The data extraction form was piloted initially using a few publications. Appropriate modifications were made before starting the full extraction process.

The data were extracted by LD using extraction table that was previously agreed with the coauthors. Data extraction was scrutinised independently by two other authors (CC and TR).

For each publication, the author, year of publication, geographical region of interest, type of study (report, discussion, consensus, etc), study design (prospective, retrospective, cross section), treatment pathway (primary, secondary or both), principal findings and key recommendations were extracted.

Most of the articles were descriptive; hence the analysis followed a narrative synthesis. This is common in reviews of very heterogeneous studies which aim to describe and scope the area of interest.^34^ Since the objective of the report was to explore options for service delivery, the review was designed to be inclusive. Publications were, therefore, not excluded based on quality criteria but were described and briefly critiqued as appropriate given the nature of the studies. We aimed to map the current literature and understand the type of evidence available in this area (ie, allergy pathways).

## Results

The database search identified 351 articles of which 158 were duplicates. Additional 12 articles were included following reference and citation searches. After consideration of the title and abstract, a further 142 articles were excluded and a total of 63 publications were screened thoroughly for their relevance to the review. Figure 1 shows a flow diagram of the papers screened, identified, retained or excluded at each stage, and the reasons for exclusion of articles as per the PRISMA guidelines.^35^

Twenty-seven publications were included in the final review which are summarised in table 1. Only three publications describe prospective data collection alongside service reorganisation.^23^ ^43^ ^52^ There were no eligible prospective, randomised controlled trials identified.

**Table 1:** Summary of characteristics of the included publications (arranged in chronological order)

Author, (year) (ref)				Level				
	Region	Type of study	Study aim	1°	2°	Salient findings	Key recommendations	Comments
Isinkaye *et al* (2016)^36^	UK	Retrospective cohort study	To ascertain what proportion of referrals to secondary care could be managed a by GP with special interest in allergy	✓		At least two-fifths of all referrals to specialists (42%) were felt to be appropriate for a GPwSI setting. There was some disagreement between reviewers re: suitability of a further 30% of the referrals Intraobserver variation was also seen (ie, reviewer changed their initial opinion on referral after seeing the letter from specialist).	GPwSI in allergy could effectively identify and manage a large proportion of referrals made to paediatric allergy specialists. This service should be introduced alongside other initiatives to improve UK allergy services.	The GP referral letters and the clinic letters from specialists were reviewed by three paediatric allergists. Generalisability of results may be an issue, although GPwSI shown to be useful by Levy *et al* as well. The authors used an agreed set of criteria for the competencies expected of a GPwSI (not provided with the paper).
Krishna *et al* (2016)^37^	UK	Report/non-systematic literature review	To discuss the potential use of telemedicine in pathways for diagnosis and management of adult allergies	✓	✓	Adult allergy services can potentially benefit from telemedicine. Various pathways are suggested. Algorithms for possible management of allergic rhinitis, urticaria and anaphylaxis via telemedicine are discussed	Authors advise that prospective studies evaluating these techniques should be planned	Telemedicine used successfully in some areas of medicine, but systematic prospective studies in allergy are lacking. There are potential issues with clinical governance and confidentiality Lack of adequately trained specialists can affect implementation of these measures.
Bousquet *et al* (2015)^38^	Europe	Introduction of prospective study using Information and communications technology (ICT) methods.	Plan for study with ICT methods in allergy services.	✓	✓	Many gaps in allergy diagnosis and management exist which could be addressed using advances in ICT. The use of Visual Analog scoring, e-allergy and MASK aerobiology apps can help in diagnosis, management and monitoring of allergic rhinitis.	The systems will be based on ARIA and International consensus of rhinitis guidelines. The use of ICT can facilitate communication between clinicians, patients, pharmacists and other stakeholders.	This project aims to use ICT systems to tackle heterogeneity in AR management across Europe. The clinical trial is being planned; but the uptake of ICT in other studies has been poor.
Conlan *et al* (2015)^39^	Ireland	Retrospective cohort study	Review of New allergy referrals to adult specialist clinic. A pilot email communication service with non-specialists.	✓	✓	A majority of patients referred to secondary care had chronic spontaneous urticaria or angioedema. Food/drug allergy or intolerance accounted for about a quarter of all referrals. The email service did not show demonstrable impact on referral numbers. It was rated as useful by those clinicians who responded to the survey.	Studies examining referral patterns can be helpful in planning services locally by targeting education of non-specialists. New models of care delivery should be tried to help ease demand on specialist allergy centres.	Study designed to help service planning locally design may be generalisable whereas findings are not. The uptake of email service was perhaps lower than expected. Also the response rate to the survey was poor (35%) which makes the usefulness of the service difficult to gauge.
Chan *et al* (2015)^40^	Hong Kong	Report	To discuss the current management of allergic disease in Hong Kong.		✓	Despite increasing demand, allergy services and training remain poor. There are dedicated allergy services in public hospitals for adults. Laboratory support for allergy and immunology is inadequate.	2 pilot ‘Hub and spoke’ centres catering for adult and paediatric allergy should be established. Training programme in paediatric and adult immunology and infectious diseases should be extended to allergy.	This is a report from the Hong Kong allergy alliance, whose members include patients, clinicians, academics, industry and other stake holders in allergy within Hong Kong.
Jutel *et al* (2013)^24^	Europe	Report/cross-section	To provide a contextual patient-centric framework based on opinion of PCPs, specialists and patients.	✓		Access to specialist services was identified as the ‘greatest unmet need’. In public health services, waiting time for secondary care is usually > 6 weeks. Current dominant model of allergy care in Europe is specialist based, but this is unsustainable.	Groups across Europe need to learn from shared experiences to generate political will to enable change to services. Patient involvement and empowerment should be strongly encouraged.	The authors of this publication belong to the EAACI Task Force for Allergy Management in Primary Care.
Jones *et al* (2013)^41^	UK	Survey/retrospective	To assess patients perception of usefulness of the secondary allergy clinic at Plymouth Hospital.		✓	A third of the patients did not find the clinic useful. Half continued to have troublesome symptoms. 10% do not feel confident about managing their allergies.	There is a need for follow-up of most patients with allergy to reinforce education. Specialist clinics should try to obtain routine feedback from patients to monitor effectiveness.	Patients who attended clinic over a 11 year period were surveyed, 36% response rate (336/933). No description of services offered or the competencies of the clinicians.
Agache *et al* (2012)^7^	Europe	Survey/cross-section	To assess the actual status of allergy management in primary care across Europe	✓		Two-thirds of PCPs do not have ready access to allergy specialists. The average waiting time to see a specialist in a public health service was more than 6 weeks. Referring patients to organ specialists is much easier than referral to an allergist.	A thorough assessment needed to understand demands on services and facilities available to PCPs. This can be used to adapt allergy pathways for primary care. To develop a structured development and information platform for PCPs.	The study was carried out by an EAACI task force. Surveys sent to the national societies of EAACI member countries and to individual members of EAACI as well as the international primary respiratory group.
Sinnott *et al* (2011)^23^	UK	Prospective planning and implementation of care pathways	Description of a pilot project undertaken to improve allergy services in the North of England.		✓	Poor training of PCPs leads to inappropriate referrals due to lack of confidence in managing allergies. Specialist services are often deluged with patients who could have been managed in primary care. Variable tariffs for allergy pose a disincentive for trusts to develop services. Postcode lottery exists especially for those with severe allergies.	Linking clinicians with an interest in allergy is a good way to improve standards and increase awareness of patient pathways. Developments should support existing service provision. Commissioners need to be educated regarding the impact of allergies. Good transition between adult and paediatric services needed.	£1.8 million pump priming for services from the DoH, UK. Getting commissioners in the NHS interested in improvement of allergy services was challenging. The project helped formation of a specialist nurse group in the region as well as a good network of clinicians interested in allergy.
Warner and Lloyd (2011)^42^	UK	Discussion/pathway development	Background for the development of paediatric allergy care pathways by the Royal College of Paediatrics and Child Health (RCPCH)	✓	✓	The pathways are aimed at commissioners, health professionals, patients, parents and carers. They aim to provide a bench-mark for service provision.	Eight pathways developed by six multidisciplinary working groups. The authors define competencies rather than criteria for onward referral, so that guidance can be applicable even when there are regional variations in service provision	Existing literature was systematically reviewed to identify ideal pathways for care and competencies required. Pathways for anaphylaxis, asthma/rhinitis, drug allergy, eczema, food allergy, latex allergy, urticaria and venom allergy were proposed.
Royal College of Physicians and the Royal College of Pathologists (2010)^21^	UK	Report from a publicly funded organisation.	Recommendations to stakeholders in allergy for provision of cost-effective improvements in allergy care. An update on changes to allergy service provision following the House of Lords inquiry (2007) into allergy.	✓	✓	Services remain poor and highly inequitable. Some progress since 2007 Additional trainees in adult and paediatric allergy were appointed. The Northwest SHA spearheaded a pilot into restructuring of allergy services. NICE had adopted a few projects for issuance of guidelines. Some areas of concern remained unaddressed including: Poor coding of allergy clinical work. Patient engagement underused Governance and training within existing services remains poor. Occupational allergy provision remains poor. Serious deficiencies found in the commissioner's knowledge of the allergy needs of the local population. PCP survey in 2009 showed that most (70%) continued to rate NHS allergy services as poor (similar to 2002 survey).	Services should join up to serve the population of a defined geographic area. Validated Patient Reported Outcome Measures (PROMs) need to be developed to evaluate the effectiveness of services. Quality Assurance schemes should be developed for clinical allergy services. Protocols and guidelines should be shared freely between centres. More allergy training should be incorporated into PCP and medical student curriculum (and all other related specialty training) Clinical services should establish good links with the local patient groups. There should be better allergen labelling.	Working party for this report consisted of clinical experts and patient representatives from all over the UK. Data from the pilot study in North West were discussed in the report. Selected publications were reviewed (non-systematic). Views from charities supporting patients with allergy also represented. Concerns expressed about the lack of funding for outcome evaluation with allergy service remodelling in the North West.
Levy *et al* (2009)^43^	UK	Prospective; no control group.	Evaluation of a PCP with special interest clinic in allergy.	✓		Two-thirds of the patients would have been referred to secondary care in the absence of this clinic. Less than 10% of those reviewed were referred onto a tertiary clinic. The clinic was estimated to have saved £13,500 in 9 months due to reduced referrals.	Second-tier clinic in primary care has the potential to be clinically effective as well as cost-effective. It encourages care in the local community and can reduce the burden of inappropriate referrals to tertiary centres.	Referrals proforma provided information on how the clinic was used by other PCPs. Consultation satisfaction questionnaire captured patient experience.
Working group of the Scottish Medical and Scientific Advisory Committee (2009)^22^	UK	Report from a publicly funded organisation.	To report on the diagnostic and clinical allergy services within Scotland	✓	✓	High burden of allergy in Scotland; 30% children and 25% adults are affected. The levels are rising for all conditions (except perhaps asthma) and services have only improved marginally since last report in 2000. There are insufficient numbers of medical specialists, trainees, PCPs, dieticians, nurses and pharmacists trained in allergy. Service is fragmented with no collaboration between primary, secondary and tertiary services. Dietetic services fragmented and patchy and are not always backed up with clinician support. Allergy curriculum in undergraduate and postgraduate medical training needs improving.	Primary care staff should have access to basic initial and ongoing training. There is a need to encourage and facilitate standardised and evidence-based practice through shared protocols and pathways. Data collection, audit and research facilities in allergy should be improved to ensure better future planning of services. Regional MCN for adult allergy and a national MCN for paediatric allergy services are needed. Involvement of voluntary sector should be encouraged to publicize the deficiencies in service.	The authors commented on the non-availability of trained specialists and the underusage of non-physician services for allergy (pharmacists, dieticians, nurse specialists). Improved motivation via incentives should be planned. PCP with special interest may be a useful resource.
Haahtela *et al* (2008)^32^	Finland	Prospective; intervention; no control group.	Nationwide allergy programme being adopted in Finland. Proposed to run between 2008 and 2018.	✓	✓	Project is currently underway. Its goals include: Prevention of allergic symptoms. Increase tolerance against allergens. Improve allergy diagnostics. Increase resources for allergy management. Decrease healthcare costs due to allergies.	For each of the five identified goals, specific tasks, tools and evaluation methods have been defined. This project is based on very close collaboration between the government, healthcare sector and non-governmental organisations. Emphasis is on tolerance and not on allergen avoidance.	The project builds on the very successful Finnish asthma model. Being followed in other countries (Norway, UK), preliminary results are expected soon.
House of Lords Science and Technology Committee, 6th report of session 2006/7 (2007)^18^	UK	Report from a publicly funded organisation	To explore the impact of allergy in the UK upon patients, society and the economy as a whole.	✓	✓	Allergy exerts a considerable social and economic burden upon the nation. There is a severe shortage of allergy specialists in the UK and the services lag far behind those of many countries in Western Europe. There are problems with data collection rendering statistics imprecise and affecting service redevelopment plans. There has been a chronic lack of training of PCPs and medical trainees in allergy, leading to problems with diagnosis and management at the primary care level. Further research into the basis of allergy is urgently needed to underpin further public health policies to address the rise the allergies. Large, tertiary centres led by allergists should be developed to ensure optimal treatment of patients with complex and severe disease and also as sources for education and training for other clinicians.	Improved education of medical practitioners to diagnose and treat occupational allergies needed. Improve undergraduate and PCP allergy training. New centres should build on existing excellence. Some specialist services can be restricted to few centres across the country. Educators and Commissioners should work together to develop generic quality assured clinical post graduate allergy courses. NICE to appraise immunotherapy and cost-effectiveness. A lead health authority should be identified by the Department of Health in order to establish a pilot tertiary allergy centre. A full cost analysis should be integral to its establishment.	This report was published by the allergy subcommittee UK House of Lords Science and Technology Committee 2007. Recommendations made for non-NHS management of allergies (eg, training teachers in managing allergic emergencies, supporting children with hay fever during school examinations, helping those with occupational allergies return to work, improving allergen food labelling, etc). Authors visited numerous national and international allergy centres of repute to compile this report.
Department of Health (2007)^44^	UK	Report from a publicly funded organisation.	Response to the report from the House of Lords Science and Technology Committee 2007.	✓	✓	No published whole system models of services for people with allergy. No data on existing skills. There are also no analyses of effects of active demand management of patient flows in allergy care. No data on allergy needs in various regions across the country.	The royal colleges should work together to set up curricula for health professional training in allergy. Health commissioners should work with local service providers to ensure best possible service planning for their catchment areas. Much clearer understanding of skills and competencies of the existing workforce needed. NICE advised to provide guidance on allergen immunotherapy.	Funding identified for an allergy centre in the North West region of England. Most of the recommendations from the House of Lords report could not be acted upon due to insufficient and unreliable data on the existing state of allergy management, according to this report.
Warner *et al* (2006)^3^	Worldwide	Cross-section; Questionnaire survey.	To define the current state of allergy training and services in the countries represented within the WAO		✓	Prevalence rates for allergies in the responding countries ranged from 7.5% to 40% (mean 22%). Number of certified allergists varied widely from 1:25 million in Indonesia to 1:16,000 in Germany. Formal certification procedure is not available for clinicians in some of the countries surveyed. In most countries, patients are first referred to organ-based specialists before being referred to allergists.	There is a very wide gap between demand and provision of allergy services worldwide. Training of medical students, general practitioners, generalists as well as system specialists who deal with allergy must improve to ensure better care provision. More tertiary level centres needed to set the standards, advance research, support training and provide expertise to primary and secondary care.	Survey sent to all WAO national society member organisations to be completed by allergists knowledgeable about services within their own countries (61 sent, 34 responses received). Data based on impressions of these experts in some countries rather than on published data.
Department of Health (2006)^45^	UK	Report from a publicly funded organisation.	Review of allergy services undertaken to fulfil Government of UK's commitment to the House of Commons Health Committee.	✓	✓	No compelling evidence on need or on quality of allergy services since relevant research lacking. Patients feel let down by a poor and often inaccessible service. Specialist services are usually not available, resulting in very long waits to see consultants where services do exist. Self-care can be particularly useful in allergy and should be promoted. Some conflict between the main two specialities offering allergy services in the UK (ie, allergy and clinical immunology).	Local commissioners need to establish levels of need for services for allergy in their health community. Educators and Commissioners should work together to create additional training spaces for doctors. Guidelines for management and care pathways should be developed by NICE.	Data obtained by review of existing literature and also by interviewing stakeholders. Highlights the difficulties in developing national strategy for allergy services without baseline data on needs and costs involved. It is important to understand the skills and competencies that exist and those that are needed from the diverse workforce to enable future development and provision of services.
El-Shanawany *et al* (2005)^46^	UK	Cross-section; Questionnaire Survey	To survey allergy services provided by clinical immunologists in the UK.		✓ ✓	Immunology centres are the only providers of tertiary allergy care for most of the UK. Consultant immunologists are likely to be providers of tertiary level allergy care in the medium and long term for the UK. Waiting times for allergy patients in these clinics were long, sometimes waiting over a year for urgent appointments. Very few centres benefitted from dietician support.	There needs to be a collaborative effort between clinical immunologists and allergists in the UK in order to improve services.	Questionnaires sent via three supra-regional immunology audit groups to the various participating immunology regional centres in the country. 17 immunology centres serving a total population of 32 million individuals responded.
Ryan *et al* (2005)^47^	UK	Discussion	To propose minimum levels of knowledge required for clinicians in order to improve standards of allergy care.	✓	✓	Self-care in allergy is problematic due to the poor access to NHS healthcare and the availability of unregulated alternate practitioners. PCPs and practice nurses could be better trained in prescribing drugs for allergy.	Intermediate care services (eg, PCP with special interest) should be developed. Pharmacists, primary care nurses and physicians could be trained in a few allergy-related techniques to vastly improve service provision.	The authors suggest that management of allergy in primary care can be improved even when specific tests and other infrastructure are unavailable. Knowledge of pharmacotherapy for allergy can help PCPs manage a majority of patients.
Department of Health (2005)^48^	UK	Report from a publicly funded organisation	Government of UK response to the House of Commons Health Committee report.	✓	✓	Good quality data on needs and services for allergy is lacking. Service models for managing allergy in primary and secondary care could be developed. Medical regulatory bodies overseeing physician and nurse training should be encouraged to increase allergy educational content during training.	Self-care should be encouraged; NHS led expert patient programme will be extended to allergy. Food Standards Agency has produced a guide for those recently diagnosed with food allergies. Local commissioners should establish need for services in their local area.	It was felt that a review of available data and research on allergic conditions is necessary in order to plan future direction of allergy services. This formed the basis for a separate report (as above).
Levy *et al* (2004)^49^	UK	Cross-section; Questionnaire survey	Understanding the views of PCPs in the UK regarding the quality of primary and secondary care for allergy.	✓		More than 80% felt that the NHS allergy care was poor. Primary and secondary care services were thought to be deficient. Very few (4%) offered skin prick tests at their practice. Most expressed concern regarding managing children with allergies. A majority were confident in the management of urticaria, allergic rhinitis, angioedema, anaphylaxis.	National education programmes should be developed for PCPs. Specialist care provision for allergy should be reviewed urgently within the NHS.	Randomly selected sample of 500 PCPs from UK General Practice register were contacted. Only 50% response rate.
House of Commons Health Committee (2004)^19^	UK	Report from a publicly funded organisation	To highlight the need for allergy service improvement in the UK	✓	✓	Primary care skill base for allergy is poor—this is compounded by weakness in secondary care sector as well. Current provision is manifestly inequitable and more allergy specialist centres are required. Better secondary care can help improve primary care knowledge and services. Paediatric services are worse than adult services —school nurse training, transition services, dietary recommendations, etc, all need improving—-specialist services can help improve school staff training in allergy by taking on leadership for this. Poor and sometimes dangerous practice exists in the independent sector. Data on waiting times are flawed, and this adversely affects service planning.	Allergy specialist centres need to be developed manned by allergists; allergists cannot be substituted effectively by other specialists. Advocated the establishment of national primary care allergy network. Ongoing training for allergy in primary care needs to improve; services should be peer reviewed. Introduction of clinical quality markers for allergy to incentivise improvement advised. PCP curriculum needs to be modified to include more allergy. Separate coding for allergy needs to be introduced (now available). Investment in allergy training required.	Health committee comprising of elected representatives. Expert interviews, statistics from published sources, submissions to panels from individuals – patients or carers (300 letters) were all used.
Royal College of Physicians (2003)^20^	UK	Report from a publicly funded organisation.	To ensure that allergy services are prioritised for improvement by commissioners and managers in the NHS.	✓	✓	Allergy incidence and prevalence is increasing but services are quite poor. Very few allergy specialists in the country and few trainees in the pipeline. General practitioners not trained to cope with the increasing demands for allergy treatment, most do not feel confident about services, but very few patients are referred to specialists, nonetheless. Few centres offer secondary care allergy; six centres UK wide offering tertiary care. Hence PCPs not sure who to refer patients to. Increasing emergency admissions for allergy. Some papers quoted to suggest specialist services may be cost-effective.	Need to have increased allergy specialists (rather than other specialists who are untrained in allergy). Important to develop regional allergy centres that can help with education, training and networking between primary and secondary care in the region (‘Hub and spoke’ configuration). More doctors should be trained to become allergy specialists. 40 new training posts in allergy will be required. Patient groups and charities must become more active and lobby for better services. There is a need for more dieticians and nurse specialists in allergy.	Working party consisting of clinical experts from all over the UK, patient representative. Selected publications reviewed (non-systematic). Other interested stakeholders interviewed, including clinicians, charities supporting patients with allergy, individual patients. Two parts to the report—one covering allergy services and recommendations for improvements and the other covering common allergic conditions and their management.
Ewan and Durham (2002)^33^	UK	Discussion	Proposal to improve NHS allergy care in the UK		✓	NHS allergy service provision is inadequate and inequitable. Estimate that there is one whole time equivalent allergist per 3.4 million population in the UK. Only six clinics in the UK offer services of full time NHS allergists.	Each of the health areas in the UK should have a regional specialist centre to provide clinical expertise and training. More training posts in allergy should be created.	Data derived from the BSACI and BAF database. Authors assume that part-time allergists provide 0.3 WTE and other specialists provide 0.1 WTE allergy work per week. This is debatable.
Ewan (2000)^50^	UK	Discussion	An overview of NHS allergy services and suggestions for improvement.		✓	There are serious deficiencies in the allergy services within the UK. Training numbers for allergy are not adequate to serve current and future demands on the specialty. Organ specialists (including immunologists) not appropriately trained for the holistic management of these patients.	Minimum of 1 regional allergy centre per region needed manned by allergy specialists and nurses, dietician. Organ-based specialists and allergists need to be appointed to more secondary level centres. There should be an increase in specialist training spaces for allergy.	Data from BSACI and BAF database as above. Recommendations as per the Allergy task force set up by the BSACI and DoH in 1998.
Brydon (1993)^51^	UK	Questionnaire; retrospective	A survey to determine the effectiveness of a nurse practitioner service.	✓		Nurse led service resulted in fewer general practitioner consultations and also a reduction in prescribed medication for allergy. Most respondents reported an improvement in symptoms. Better results seen in patients who were followed up for longer.	Using nurse led services in primary care can be cost saving. There could have been a recruitment bias/criteria for choosing a section of patients not made explicit.	Bespoke postal questionnaire before and 9 months after appointment with the nurse. Responses compared with patient notes from PCP.

BAF, British Allergy Foundation; BSACI, British Society of Allergy and Clinical Immunology; DoH, Department of Health (UK); EAACI, European Academy of Allergy and Clinical Immunology; MCN, Managed Clinical Network; NHS, National Health Service (UK); NICE, National Institute of Health and Care Excellence, UK; PCP, Primary Care Physician; PROM, Patient Reported Outcome Measures; WTE, Whole Time Equivalent; WAO, World Allergy Organisation.

Level: 1° (primary) refers to care delivered by primary care physicians, nurses and other practitioners who are non-specialist and offer services in the home or community.

2° (secondary) services refer to those provided in hospitals by clinicians (doctors or nurses) deemed to have specialist training and knowledge relevant to the management of the condition.

Seven of the publications discussed allergy services in other parts of the world,^3^ ^7^ ^24^ ^32^ ^38–40^ whereas the rest are focused specifically on services in the UK. Of the 19 UK papers, 8 are reports published by governmental organisations discussing the state of allergy services in the UK.^18–22^ ^44^ ^45^ ^48^ One of these reports provides a brief overview on aspects of allergy services in other European countries (Germany and Denmark).^18^ Another summarises experiences following the establishment of a pilot allergy service in the North West of England.^23^

Reorganisation of primary care was addressed by seven articles, secondary care services were the focus of six publications, whereas four papers discuss both levels of care. The eight government reports discuss all aspects of service delivery (table 1). Three studies discussed the use of digital technology-based interventions for allergy,^37–39^ one of these retrospectively evaluated such a service.^39^ Findings, statements and recommendations about allergy service pathways from the included papers are reported in table 1 and are synthesised thematically.

### Primary care services

#### PCPs in allergy service delivery

PCPs are the first-line providers of healthcare in most countries around Europe.^24^ They are well placed to provide diagnosis and management of mild and most of the moderate allergic conditions as well as to refer individuals with complex and severe allergies to specialist services.^24^ Many publications have identified that the training offered to PCPs in allergy currently is inadequate.^18–23^ ^47^ ^49^ The current inadequacies in training and the need for more information and training for PCPs in allergy were reinforced in studies reported from Scotland, Italy and Spain.^7^

It was argued in the two European publications that a model of care which is centred on specialists or consultants is untenable in allergy.^7^ ^24^ In public-funded health systems such as the UK where PCPs assess and manage the majority of patients, the burden placed by allergy and related conditions on primary care could be significant. For example, it was estimated that allergy accounts for 8% of all general practice consultations in the UK and that up to 11% of the total drugs budget is spent on allergy-related medication (including asthma and eczema).^18^

One particular article mentioned the lack of access to secondary services as allergy's ‘greatest unmet need’.^7^ Referral times to specialists vary considerably across Europe from over 3 months in some tax-funded health systems^7^ ^20^ ^22^ ^40^ to as little as 1 week when specialists can be accessed privately.^7^ Across Europe organ specialists are generally more readily accessible to PCPs than allergists.^7^ In a UK-based survey of over 480 PCPs, 81.5% of the 240 PCPs who responded felt that the NHS allergy services were poor and 80% felt that secondary care provision was inadequate.^49^ These practitioners admitted to being especially anxious about treating children with food allergies, although most felt quite confident about managing common allergic conditions such as anaphylaxis, urticaria, allergic rhinitis and drug allergy.^49^

#### PCPs with an interest in allergy

Two publications specifically discussed a second tier service for allergy within primary care.^7^ ^53^ Such an arrangement was also proposed by the House of Lords report.^18^ In the UK, a prospective evaluation of patients referred to a General Practitioner with Special Interest (GPwSI) in allergy revealed that the services were well received, reduced the levels of secondary care referral and had a potential for cost savings.^43^ Further, PCPs in this study referred patients more readily to the GPwSI than to secondary care.^43^ However, establishing these services would need a well-defined process of accreditation and specialist mentorship^24^ which may be difficult to achieve in most countries given the current severe shortage in the availability of specialists across Europe.^3^ ^24^

#### Non-physician services in primary care

Non-physician services for allergy were specifically discussed by six publications in this review.^18^ ^20^ ^22^ ^44^ ^47^ ^51^ Most of the articles discuss the underusage of these professionals in allergy and suggest that there is a scope for better training of nurses, pharmacists and dieticians in allergy. Depending on the extent of training and the competencies achieved, nurses could be involved with testing, diagnosis and management of patients with allergy.^47^

Some authors felt that pharmacists could, if adequately trained and sufficiently supervised, provide information to patients regarding techniques for using devices such as nasal sprays, eye drops, epinephrine auto-injectors as well as inhalers for allergy and related conditions.^18^ ^47^ They could help patients choose over the counter medication for allergy judiciously. They can also be trained to advice individuals on the need for consultation with their PCP, where appropriate.^22^ The House of Lords committee suggested that pharmacists should be formally trained in allergy to ensure that good quality advice on allergy medication can be provided to all patients.^18^ This committee also reported concerns from clinicians regarding availability of unvalidated tests over the counter for allergies in some establishments.^18^ There are, however, no publications to-date formally assessing the role of pharmacists in the diagnosis and management of allergy.

#### Barriers to providing optimal allergy care in the primary care sector

Several authors were concerned that PCPs do not receive structured instruction in allergy during their training, and very few are familiar with guidelines for the management of allergic disease.^7^ ^20–22^ ^33^ The House of Commons health committee highlighted the lack of allergy knowledge in primary care as “…one of the principal causes of distress to patients”.^19^ Some articles have specifically highlighted the significant gaps in allergy training at the undergraduate and postgraduate levels, as well as inadequate continuing medical education programmes for PCPs in allergy.^20^ ^21^ ^24^ This was identified as leading to inappropriate referrals to a range of specialists,^23^ lack of engagement with secondary care services for allergy, delays in diagnosis and starting appropriate management^20^ and, sometimes, to inappropriate management.^33^ All these issues resulted in poor patient experience and also cause a significant wastage of scarce healthcare resources.^20^ ^21^ A retrospective review of the patients at a secondary care allergy clinic in Sussex showed that at least 42% of patients were referred for conditions that could have easily been managed in primary care, had the PCPs been appropriately trained.^36^ An Irish study also suggested that increasing awareness of common allergic conditions among PCPs can significantly reduce referrals to specialists.^54^ This suggestion was reinforced in UK government reports^19–22^ and other studies.^23^

In most countries, the lack of leadership and support offered by a stable, well-staffed specialist service was identified as one of the main barriers to improvement of primary care services.^7^ ^18–21^

### Secondary care services

#### Availability of specialist services

A publication by the World Allergy Organisation (WAO) has suggested that there is a great degree of heterogeneity in access to specialist allergy services across the world.^3^ ^40^ Experts point out that while there has been very little increase in availability over the last few years, the demand for specialist allergy services has been steadily increasing.^21^ For example, the number of certified allergy specialists per head of population range from 1:25 million (in Malaysia) to about 1:2 million (in the UK) and 1:16 000 (in Germany).^3^

Heterogeneity in specialist training has also been highlighted^3^ ^33^ with only a few countries providing certified courses to practitioners in allergy. A worldwide study by the WAO showed that paediatric allergy services are particularly underserved and children with allergic problems are often managed by general paediatricians with or without formal allergy training.^3^ This study also found that in many countries children may be managed by specialist adult physicians without appropriate paediatric training.^3^ Specialist training pathways for allergy vary markedly worldwide. In countries such as the UK, formal certification procedures in either allergy alone or in a combination of allergy and immunology exist. Similarly, in the USA, allergists/immunologists should have passed a professional examination taken after 2 years of structured specialty training. In other countries, allergy may be included as a subspecialty in general internal medicine or paediatrics training.^3^ In Germany, for example, allergology is considered a subspecialty of dermatology.^18^ In the UK, the British Society for Allergy and Clinical Immunology (BSACI) has estimated that 90% of secondary care in the UK is provided by allergists and immunologists.^45^ A study carried out in the UK has shown that immunologists, who have formal training in allergy, provide allergy care to 32 million individuals in the UK.^46^ Some authors have pointed out that immunologists are indeed the sole providers of allergy services in parts of the UK.^44^ ^46^ Other specialists such as those with primary qualifications in ENT, respiratory medicine or dermatology also contribute to the delivery of allergy services in many countries^3^ including about 10% of the total secondary care for allergy in the UK.^45^ Even if this broad definition of allergy specialists were to be accepted, many experts feel that allergy services remain inadequate in most countries in the face of increasing demand for these services.^3^ ^19^ ^40^

#### Specialist centres for allergy

Some authors propose the ‘hub and spoke’ model^18–20^ ^40^ which involves the establishment of supraregional tertiary allergy centres (or Hubs) which can support regional secondary and primary care centres (the so-called spokes) for delivery of specialist services. A few suggested that these centres should be manned by consultant adult and paediatric allergists, nurse specialists as well as adult and paediatric dieticians while providing facilities for training at least two specialist registrars in allergy.^20^ Others felt that these should be multispecialist centres (eg, chest physician, dermatologist, ENT specialist, paediatrician in addition to an allergist or clinical immunologist) that are built on existing expertise of the local area and serve as ‘clusters of expertise’.^18^ In some countries, these centres would typically be university hospitals which would receive referrals only from specialists.^18^

Whatever their composition, most agreed that these centres could serve to educate and support primary and secondary care physicians in the region.^18–20^ It was suggested that they had a potential to serve as centres of excellence for adults and children with complex and severe allergies; establish a good, working network between organ-based specialists, generalists and allergists and serve to improve the overall provision of allergy services in the region.^18^

Some experts point out that the existing shortage of specialists in allergy would be a barrier to the development of such centres.^21^ ^50^ A pilot study carried out in the North West region of England found that developing large tertiary centres would not be practical in regions with large cities in close proximity to one another.^23^ They may not be cost-effective for many regions within the UK^21^ and perhaps, Europe.

The House of Commons health committee has pointed out that there are no clear data to suggest that specialist centres improve clinical outcomes in allergy management.^19^ ^41^ Indeed, even in countries like Germany with a relatively high proportion of allergy specialists per 100 000 population, the numbers of emergency admissions for allergy remain high.^3^ The North East England pilot study found that the lack of confidence among general practitioners while dealing with patients with allergy led to poor referral practices.^23^ As a consequence, management of simple conditions took up a disproportionate amount of specialist time and resources while individuals with complex allergies faced long waiting lists as well as inappropriate referrals to other specialists.^23^

### Future direction for services

While efforts are being made to improve allergy education at the undergraduate and postgraduate levels, there has been a focus also on the improvement of training of current practitioners. The Royal College of Paediatrics and Child health has developed care pathways for children which define core competencies for all those involved in managing these conditions and are freely accessible online.^42^ These are UK based but potentially can be modified to suit other countries. Such pathways embrace the current heterogeneity in service delivery while attempting to raise standards.

The ‘Hub and spokes’ model was trialled in the UK with mixed results, which was specifically discussed in a report.^23^ The authors suggested that new services should be tagged onto existing pathways and also stated that a care model of visiting specialists in secondary centres would be more welcome in some areas than the establishment of large tertiary centres.^23^ It was also suggested that models of good care can vary from one region to another.^21^ ^23^

There have been recent publications regarding the use of digital technology in the provision of allergy services.^37–39^ One addresses the use of telemedicine in improving communications between primary and secondary care in order to improve adult allergy pathways within the NHS;^37^ whereas another makes a case for clinical trials using information communication and technology (ICT) in management of allergic rhinitis in Europe.^38^ A publication from Ireland reported on the use of an email communication system, which received an average of only four enquiries per month over a 12-month period. Although it was rated useful by 100% of the responding non-specialists (response rate of 35%), this communication system did not reduce referrals to the specialist allergy services.^39^

There has been a lot of interest lately in the ‘Finnish model’ of service reorganisation. This re-structuring exercise takes inspiration from the successful interventions for asthma in Finland.^32^ While acknowledging the differences between asthma and allergy and emphasising the need to understand and improve tolerance to allergens, the architects of this model hope to use the existing asthma infrastructure to improve services for allergy sufferers. They suggest that increased initial outlay aimed at preventing allergies and changing attitudes towards health alongside improving service delivery can reduce the cost and burden of allergic disease in the future.^32^ The results of this experiment are currently awaited.

## Discussion

### Principal findings of the review

This systematic review aimed to identify and discuss various pathways that are relevant to the delivery of allergy services. There were large gaps in the literature pertaining to services in countries with high rates of allergy (such as Australia, New Zealand, USA)^1^ ^5^ as well as very populous regions of the world including China, India, Brazil and the whole of Africa. In addition, there was a lack of well-designed studies in this area with only three prospective studies identified.^23^ ^32^ ^43^ None of the studies included a control group. Two of these publications^23^ ^32^ describe service reorganisation on a large scale with direct involvement of the relevant health ministries.

There is clear evidence from the literature that allergy services across the world have not kept up with rising demand. The ‘allergy epidemic’^13^ has surprised unprepared health systems globally. There has been failure on the part of governments and fund holders to acknowledge the rapid rise in allergies. Given that there are no signs of abatement in the observed increase in allergies worldwide,^2^ it is conceivable that the demand on services is set to increase even higher over the next few years. The psychosocial impact of these conditions is often overlooked. For example, atopic individuals experience significantly worse memory and cognitive ability during allergy season.^55^ Children with eczema report higher levels of anxiety and depression.^56^ In addition, these conditions currently place an inordinate financial burden on healthcare services.^29^ ^57^ ^58^ Urgent and effective measures are therefore needed to cope with the problem.

About three-quarters of the eligible publications in this review (18/23) are from the UK which suggests that there has been a lot of interest here in investigating the extent of the supply gap in allergy services over the last 15 years. It is striking however, that while most of these reports describe the problems with service delivery and suggest some solutions, none seem to have addressed the problem in a structured manner. There has been no response to the UK Department of Health's request for reliable baseline data on needs of the population; costs involved in service reorganisation; and the skills and competencies of the existing workforce in order that future services can be planned.^44^ ^45^ ^48^

Primary care services are key to optimal management of allergy. Appropriate management after good history taking and specific testing can easily be achieved in primary care for a majority of patients. Referral to specialist centres can be limited to only complex patients needing multidisciplinary input or those that need desensitisation therapy. However, a UK survey has shown that PCP confidence in managing allergies in children^49^ and initiating referrals appropriately is limited. While PCPs in this particular survey felt confident about managing adults, studies have shown that most individuals referred to secondary care could have been managed effectively in primary care.^23^ ^54^ ^59^ This serves to highlight the inadequate training received by PCPs in allergy at undergraduate and postgraduate levels. This leads to not only poor patient experience and outcomes but is also more expensive to the health service providers.

Owing to lack of specialists in allergy, patients are often referred to specialists who can, perhaps, only deal with individual manifestations of allergy (eg, respiratory physicians for allergic asthma; ophthalmologists for allergic eye disease). Organ-based specialists play a very important role in the management of allergic disease. Indeed, in some instances (eg, children with very severe disease), their input is essential. However, specialists in allergy can provide clinically effective and potentially cost-effective services by intervening across several of these conditions for most patients.^20^

Scarcity and inequity of specialist allergy services is a recurring theme in many articles worldwide. Although numerous publications have made a compelling case for more specialist centres,^3^ ^18–21^ ^33^ these have not been forthcoming. Many factors appear to contribute to this apparent inertia,^21^ the important ones being lack of adequate central funding to increase training numbers for specialists, lack of interest in allergy services among fund holders,^23^ lack of clarity regarding the role of various specialists involved.^21^ Another important issue is the lack of formal training programmes in allergy in many countries.^3^ This not only blights the care of individuals with allergy in these countries, but also prevents the specialty being taken seriously by decision makers. In the case of the UK, lack of clinical codes to measure allergy activity and disagreements between the two main specialist groups that provide allergy services (allergists and immunologists) are also important issues.^48^ Further, in the UK, the lack of specialist services and poor referral practices within primary care have resulted in unreliable waiting list data, which are often used as a surrogate marker for need within the NHS.^44^ This has proved to be a barrier for further investment in services.^48^

It should be noted that there are no published data that support the success of large, tertiary centres. Nevertheless, it is conceivable that centres which treat large volumes of individuals will provide better outcomes for complicated patients.^60^ However, the lack of confidence among general practitioners while dealing with patients with allergy leads to poor referral practices leading to long waiting lists as well as inappropriate referrals to other specialists.^23^

There have been many encouraging advances in allergy service reorganisation in the UK and beyond. New multiconsultant allergy centres were created in the North West of England as per the recommendations of the House of Lords report into allergy services.^18^ This service development encountered many barriers including non-engagement of local commissioners, non-availability of appropriately trained staff and poor coding practices.^23^ Nevertheless, the project was successful in improving networking among specialists across the region, improved clinical governance including audit, better regional education programmes for clinical staff and patients in allergy.^23^ There was an opportunity during the course of this project to prospectively collect data on patient experiences and outcomes, which was unfortunately missed.

The heterogeneity in specialist training across Europe is also being addressed with the introduction of the European Examination in Allergology and Clinical Immunology since 2008 by the European Academy of Allergy and Clinical Immunology (EAACI). The aim of this examination is to “raise standard of allergology and clinical immunology in Europe” and to “facilitate the exchange of young people trained in Allergology and Clinical Immunology” in Europe.^61^

The Finnish allergy model is based on the very successful restructuring of asthma care in Finland^62^ and is now being adapted to the management of other chronic conditions.^63^ In Finland, the model has been altered to incorporate the complex and heterogeneous nature of allergy but it essentially builds on the existing infrastructure developed for the asthma programme.^32^ The Finnish allergy plan is an ambitious project that aims to reduce the burden of allergic disease by improving tolerance and reducing the emphasis on allergen avoidance in affected individuals. The objective is to help alleviate the psychosocial aspects of allergy while improving services provided to these persons.^32^ Aspects of this plan have also been adopted by Norway^64^ and by a health authority in North West London as well as in Sheffield.^65^ Preliminary results from the London project are very encouraging.^31^ ^66^ ^67^ More data are awaited to ascertain whether the project has been successful and also if this success can be emulated in other regions.

### Strengths and limitations of the review

The strength of this review is that it provides a systematic and comprehensive look at the reported current provision of allergy services across the world. There are some limitations to this review, mainly due to paucity of information from most countries, including some with relatively high allergy incidence and prevalence, regarding available services. Most of the literature is UK based and hence generalisability of data to other countries, especially those without publicly funded health systems may be limited. In addition, there were very few well-planned prospective studies and no controlled studies in this area. Most of the included studies had little empirical data, and therefore a formal quality assessment of the publications was not carried out. Studies not reported in the English language were excluded.

### Strengths and limitations in relation to other studies

This paper is the first to comprehensively review all the published reports and journal articles on allergy services. Our review, in concurrence with a previous UK review,^45^ found that prospective studies in the area were lacking and that there were no data objectively comparing different levels of service delivery (eg, primary care vs secondary care).

## Conclusions

There is a consensus that allergy services across the world are inadequate to meet the rising demand. There is a high degree of heterogeneity and inequity in the availability of services across the world. Untreated or poorly treated allergic conditions can have a high psychosocial impact on individuals and can place a substantial economic burden on healthcare services. Allergy training is not adequately provided in the current undergraduate and postgraduate medical curricula, which is adversely affecting patient care at all levels, especially in primary care. Primary care services are affected by poor training of practitioners and by poor access to specialists. Specialist services are hampered by the non-availability of appropriately trained personnel and poor referral practices from primary care (where applicable) which lead to long waiting lists and poor overall patient care. There is currently no clear consensus on how services should be structured although the Finnish model of service reorganisation has shown significant promise. Political engagement and patient empowerment are important to the success of these projects.

### Future research

There is a need for data on service pathways from across the world, especially from countries with a high burden of allergic disease so that the extent of the problem can be identified and lessons may be learnt from successful models. Prospective data aimed at estimating the costs and outcomes of service pathways are especially important. To ensure that a service is successfully re-organised, it is important to understand the needs of the local population, their preferences for services and to estimate costs and benefits of the possible service pathways. This literature review forms part of a wider project which aims to achieve these objectives for the population of the West Midlands region of the UK.
