# Supplementary material for: Systematic review of pathways for the delivery of allergy services
Source: BMJ Open. 2017 Feb 7;7(2):e012647. doi: 10.1136/bmjopen-2016-012647 (PMC5306521; doi:10.1136/bmjopen-2016-012647)
Supplement: supplementary file 2 [file bmjopen-2016-012647supp_Search-Strategy.pdf]

Appendix S2: Medline databases (OVID and EMBASE)

1. allerg*.hw,sh.
2. eczema.hw,sh.
3. 1 or 2
4. (care adj pathway*).mp.
5. 3 and 4
6. (servic* adj pathway*).mp.
7. (allerg* adj service*).mp.
8. 5 or 6 or 7
9. conference abstract.pt.
10. 8 not 9
11. limit 10 to human
12. limit 11 to english
13. Remove duplicates from 12
